# Enzymatic activity and brine shrimp lethality of venom from the large brown spitting cobra (*Naja ashei*) and its neutralization by antivenom

**DOI:** 10.1186/s13104-020-05167-2

**Published:** 2020-07-06

**Authors:** Mitchel Otieno Okumu, James Mucunu Mbaria, Joseph Kangangi Gikunju, Paul Gichohi Mbuthia, Vincent Odongo Madadi, Francis Okumu Ochola

**Affiliations:** 1 Department of Public Health, Pharmacology, and Toxicology, University of Nairobi, Nairobi, Kenya; 2 Department of Medical Laboratory Science, Jomo Kenyatta University of Agriculture and Technology, Nairobi, Kenya; 3 Department of Veterinary Pathology, Microbiology, and Parasitology, University of Nairobi, Nairobi, Kenya; 4 Department of Chemistry, University of Nairobi, Nairobi, Kenya; 5 Department of Pharmacology and Toxicology, Moi University, Eldoret, Kenya

**Keywords:** Snake venom phospholipases A_2_, Brine shrimp lethality assay, Snake venom toxicity, *Naja ashei*, Brine shrimp, *Artemia salina*, Probit analysis, LC_50_, EC_50_, MPC_50_

## Abstract

**Objective:**

*Naja ashei* is a snake of medical importance in Kenya, Ethiopia, Somalia, Uganda, and Tanzania. Little is known about the enzymatic (snake venom phospholipases A_2_; svPLA_2_’s) and toxic (lethal) activities of *N. ashei* venom and crucially, the safety and capacity of available antivenom to neutralize these effects. This study aimed to determine the enzymatic and toxic activities of *N. ashei* venom and the capacity of Indian and Mexican manufactured antivenoms to neutralize these effects. The protein content of the venom and the test antivenoms were also evaluated. A 12-point log concentration–response curve (0.5–22.5 µg/mL) was generated on an agarose-egg yolk model to predict the svPLA_2_ activity of the venom. The toxicity profiles of the venom and antivenoms were evaluated in the brine shrimp lethality assay. Lowry’s method was used for protein estimation.

**Results:**

Low and intermediate concentrations of the venom exhibited similar svPLA_2_ activities. The same was true for concentrations > 15 µg/mL. Intermediate and high doses of the venom exhibited similar mortalities in brine shrimp and test antivenoms were generally non-toxic but poorly neutralized svPLA_2_ activity. Mexican manufactured antivenom had lower protein content but neutralized venom-induced brine shrimp lethality much more effectively than Indian manufactured antivenom.

## Introduction

Snakebite may be the World’s biggest hidden health crisis [1, 2]. Estimates from the World Health Organization suggest that up to 2.7 million people are envenomed by snakes yearly and close to 140,000 die [3]. Non-fatal envenoming may also result in permanent disabilities including blindness, extensive scarring, contractures, restricted mobility, and amputations [4].

**Fig. 1:**
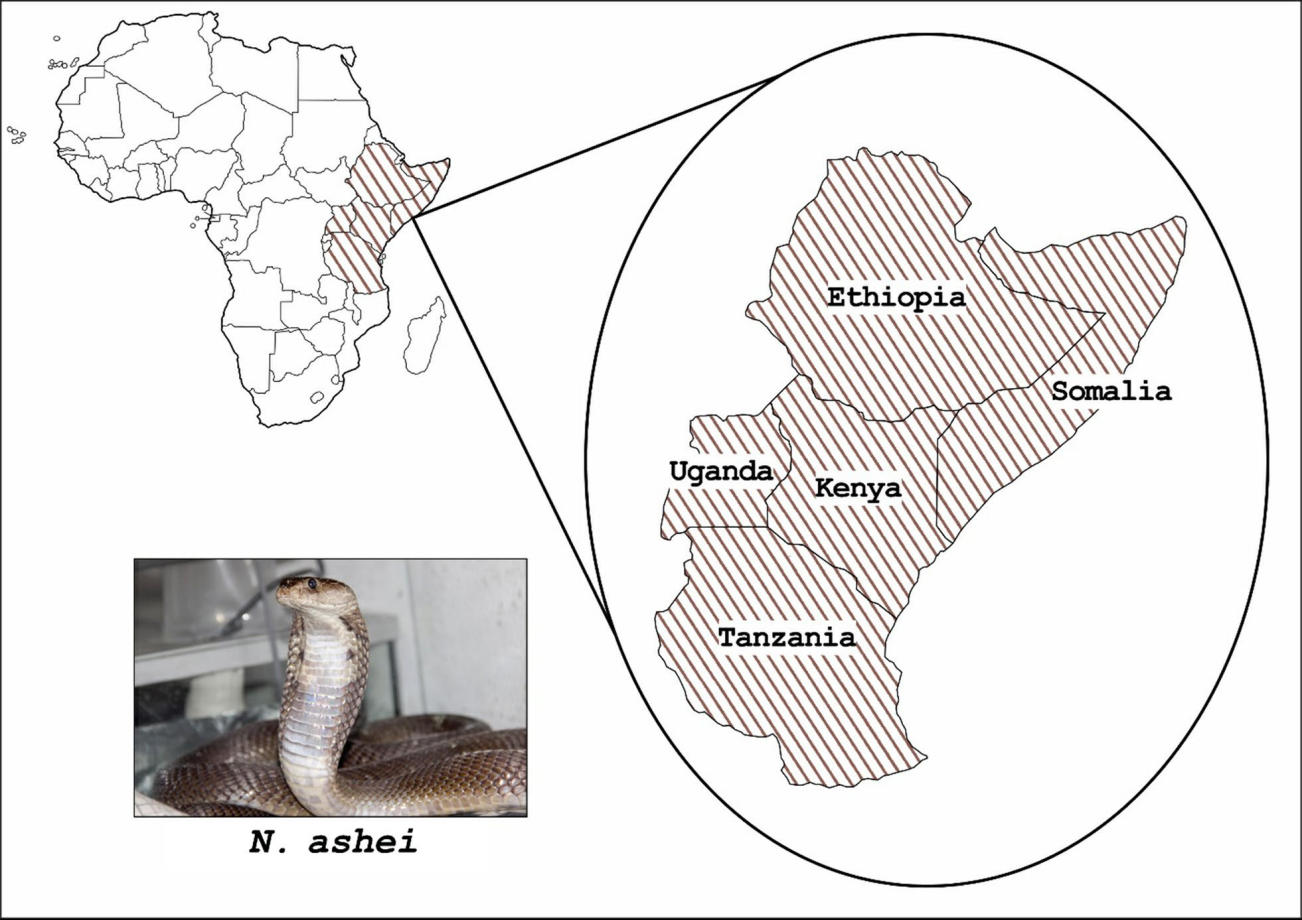
Distribution of *Naja ashei* in Africa (Source: Image of *Naja ashei* adapted from Wikimedia Commons (Lika Ivanova: https://commons.wikimedia.org/wiki/File:NajaAshei.jpg))

*Naja ashei* is a category 1 snake in Kenya, Ethiopia, Somalia, and Uganda and a category 2 snake in Tanzania [5] (Fig. 1). Category 1 snakes are highly venomous and result in high levels of morbidity, disability, or mortality [5]. Category 2 snakes are highly venomous, may cause morbidity, mortality, disability, or death but lack data to implicate them in snakebite [5].

Over the last decade, there has been a lot of interest in *N. ashei* [6–11]. The skull structure [9], mitochondrial DNA [10], composition, antiproliferative, and antibacterial properties of *N. ashei* venom have been reported [6–8, 11]. However, there has been little focus on the enzymatic, and lethal effects of this venom and the capacity of antivenoms to neutralize them. This study aimed to fill this gap by determining the enzymatic and toxic activities of *N. ashei* venom and the capacity of antivenoms to neutralize them.

## Main text

### Materials and methods

#### Snake venom and antivenom

Venom was extracted from specimens of wild-caught *N. ashei* maintained at the Bioken Snake Farm in Kenya (Table S1); 10.6084/m9.figshare.12562055.v1. Collected venom was snap-frozen and stored at − 20 °C. Reconstitution was done in phosphate-buffered saline (PBS) at the time of use. Antivenoms were sourced from hospitals in Kisumu County, Kenya. See (Table S2); 10.6084/m9.figshare.12562055.v1.

#### Animals (brine shrimp)

Brine shrimp eggs were commercially sourced from *yourfishstuff* (Borough of Lebanon, New Jersey, USA; Batch number; X001M8M5IZ). They were hatched at the Department of Public Health, Pharmacology, and Toxicology, University of Nairobi, and brine shrimp larvae were used for experiments.

#### Protein content determination of the venom and antivenoms

Lowry’s method was used [12]. An eight-point calibration curve (0.05–2 mg/mL) was developed using bovine serum albumin (BSA) as standard. Absorbance was recorded at 660 nm and the protein content of samples was inferred from the standard curve. See 10.6084/m9.figshare.12562136.v2.

#### svPLA_2_ activity of venom

The methods of Haberman and Hardt and Felix Silva et al. were used [13, 14]. Wells were made on sterile petri dishes containing agarose egg-yolk media (1:3 v/v egg yolk: PBS+ 125 µL of 0.1 mM CaCl_2_) prepared in a laminar flow cabinet. 10 µL of previously incubated (37 °C, 1 h) and serially diluted venom was discharged into the wells and incubated for 24 h at 50 °C. Carbol Fuchsin was used to visualize the halos which were measured by Vernier calipers. PBS was used as a negative control. Triplicate determinations were made and the least amount of venom required to elicit a 50% svPLA_2_ response (MPC_50_) was determined by regression analysis.

#### Neutralization of the svPLA_2_ activity of venom by antivenom

The method of Iwanaga and Suzuki 1979 was used [15]. 10 µL of a 2MPC_50_ dose of venom was mixed with 20 µL of various doses of test antivenoms (25–400 µg/mL) in 96-well plates for 5 min on a microplate shaker. The plate was incubated at 37 °C for 20 min, 200 µL of the substrate (1.1% egg yolk suspension in 0.1 M PBS adjusted to pH 8.1 and 125 µL 0.2 mM CaCl_2_) was added to all wells, incubated at 37 °C and the change in absorbance of the substrate (0 to 30 min) was determined spectrophotometrically at 620 nm [15]. Triplicate determinations were made and the least amount of antivenom required to reduce svPLA_2_ activity by 50% (EC_50_) was determined by regression analysis.

#### Determination of the brine shrimp lethality of venom, antivenom, and controls

The method of Meyer et al. was used [16]. Ten, 48-h old brine shrimp larvae were transferred from a hatching trough to 5 mL sample vials. Aliquots (5, 50 and 500 µL) of 5 mg/mL stock solutions of the samples (venom/antivenom) were pipetted into the vials and made up to the mark using 38.5% w/v marine salt solution to make 10, 100, and 1000 µg/mL sample concentrations respectively. PBS and vincristine sulphate were used as negative and positive controls respectively. Quintuple determinations were made and surviving larvae were counted after 24, 48, and 72 h. LC_50_s of samples were calculated by probit analysis. LC_50_ was defined as the least concentration of samples which resulted in 50% mortality of brine shrimp [17].

#### Neutralization of venom-induced lethality

The WHO (World Health Organization) protocol on venom neutralization by antivenoms was used with modifications [5]. Varying doses of the antivenoms (25–400 µL of 100 mg/mL) were mixed with a 2LC_50_ dose of venom. The venom/antivenom mixtures were incubated at 37 °C for 30 min, added to vials containing brine shrimp larvae and surviving larvae were counted after 24, 48, and 72 h. The median effective concentration (EC_50_) of the antivenoms was determined by regression analysis and was defined as the minimum amount of antivenom (in µL) that was required to neutralize 1 mg of venom [5].

#### Statistical analysis

Venom concentrations were converted to log_10_ (x-axis) and mean responses were converted to percentages (y-axis). The concentration of venom responsible for 50% svPLA_2_ activity (MPC_50_) was predicted by regression analysis (SPSS v20). Mortalities were converted to probits and regressed against the log concentration of venom (MS Excel 2013) [18, 19]. Analysis of variance and Tukey’s post hoc test (*p *< 0.05) was used to evaluate dose-dependent differences in svPLA_2_ activity and brine shrimp lethality. Meyer’s and Clarkson’s criteria were used to infer the toxicity of substances tested in the brine shrimp lethality assay [16, 20].

### Results

There was no significant difference (*p* > 0.05) in the svPLA_2_ activity of venom doses ranging from 0.5 to 8 µg/mL (Table 1). See 10.6084/m9.figshare.12562163.v2. The same was true for doses > 15 µg/mL (Table 1). There was a positive linear relationship between the log concentration of venom and the  %svPLA_2_ activity (Figure S1); 10.6084/m9.figshare.12562175.v3. The correlation was significant; r(65) = 0.804, *p *< 0.05, regression equation was ŷ = 26.339x + 51.906, and r^2^ = 0.646; that is, 64.6% of the variance in the  %svPLA_2_ activity was predictable from the log concentration of venom. Based on this regression model, the MPC_50_ was found to be 0.847 µg/mL.

**Table 1:** The dose–response relationship of svPLA_2_ in *Naja ashei* venom

Concentration of venom (µg/mL)	Log_10_ concentration	Mean (SD) (n = 6)	%svPLA_2_ activity
0	–	0.0 (0.0)	0.0 (0.0)
0.5	− 0.3010	9.5 (0.5)^a^	49.3 (3.3)^a^
1.0	0.0000	10.0 (0.6)^a^	52.0 (5.6)^a^
2.0	0.3010	11.8 (1.6)^ab^	61.5 (8.9)^ab^
4.0	0.6021	12.2 (1.0)^ab^	63.3 (8.1)^ab^
8.0	0.9031	12.0 (0.9)^ab^	62.5 (8.3)^ab^
10.0	1.0000	13.8 (1.6)^b^	72.0 (10.6)^bc^
12.5	1.0970	14.4 (2.4)^bc^	75.4 (17.2)^bcd^
15.0	1.1760	17.1 (2.1)^cd^	88.6 (10.4)^cde^
17.5	1.2430	18.8 (1.5)^d^	97.0 (3.4)^e^
20.0	1.3010	16.7 (1.2)^cd^	86.3 (3.7)^cde^
22.5	1.3522	17.7 (0.4)^d^	91.8 (7.6)^de^

Means with different superscripts along the columns are significantly different from each other at *p *< *0.05*(ANOVA and Bonferroni post hoc test)

*svPLA*_*2*_ snake venom phospholipase A_2_

There was a negative linear relationship between the log concentration of the venom + antivenom I mixture and %svPLA_2_ activity (Figure S2); 10.6084/m9.figshare.12570617.v1. The correlation was significant r(14) = 0.669, *p *= *0.006* and regression equation was ŷ = − 44.792x + 154.164. Based on this regression model, 1 mL of antivenom I neutralized 0.08 µg of svPLA_2_.

There was a negative linear relationship between the log concentration of the venom + antivenom II mixture and %svPLA_2_ activity (Figure S3); 10.6084/m9.figshare.12571100.v1. The correlation was significant r(14) = 0.772, *p *= 0.001 and regression equation was ŷ = − 44.706x + 162.226. Based on this regression model 1 ml of antivenom II neutralized 0.05 µg of svPLA_2_.

Test antivenoms were generally non-toxic (Table 2), *N. ashei* venom was more toxic than vincristine sulphate (positive control) after 24 h but vincristine sulphate was more toxic than venom after 48 and 72-h (Table 2). See also 10.6084/m9.figshare.12571547.v1, 10.6084/m9.figshare.12571283.v1, and 10.6084/m9.figshare.12571283.v3. There was no significant difference (*p* > 0.05) in the % mortality of brine shrimps exposed to 100 µg/mL or 1000 µg/mL of venom after 24, 48, and 72 h. See 10.6084/m9.figshare.12562199.v2.

**Table 2:** The brine shrimp cytotoxicity profile of *Naja ashei* venom, two commercial antivenoms, and vincristine sulphate (standard cytotoxic agent)

Sample	Duration of exposure	Mortality per test dose			LC_50_ (µg/ml)	Toxicity	
		10 µg/ml	100 µg/ml	1000 µg/ml		Meyer’s toxicity index [16]	Clarkson’s toxicity index [20]
Vincristine sulphate (positive control)	24	0	30	46	171.83	Toxic	Highly toxic
	48	35	50	50	2.10	Toxic	Highly toxic
	72	50	50	50	All died	Toxic	Highly toxic
Antivenom I	24	0	0	0	No mortality	Non toxic	Non toxic
	48	11	8	29	2346.23	Non toxic	Non toxic
	72	13	9	29	5268.05	Non toxic	Non toxic
Antivenom II	24	0	0	0	No mortality	Non toxic	Non toxic
	48	11	8	13	599,484,250.30	Non toxic	Non toxic
	72	12	11	17	1622.89	Non toxic	Non toxic
*Naja ashei* venom	24	0	48	50	63.02	Toxic	Highly toxic
	48	26	50	50	4.73	Toxic	Highly toxic
	72	40	50	50	0.15	Toxic	Highly toxic

One milliliter of antivenom II neutralized 0.207 mg of venom in the brine shrimp lethality assay but antivenom I was ineffective. See 10.6084/m9.figshare.12570620.v2. The mean protein content of the venoms and antivenoms was significantly different from each other. See 10.6084/m9.figshare.12573425.v1.

### Discussion

*Naja ashei* venom is yet to be included in immunizing mixtures of commercially available antivenom. In this study, we have demonstrated that Mexican and Indian manufactured antivenoms poorly neutralized the svPLA_2_ activity of this venom. We have also established that only the Mexican manufactured antivenom was effective in neutralizing the toxic effects of this venom. These observations highlight the limited efficacy of imported antivenoms in neutralizing key toxins in the venom of a snake associated with many bites in East Africa [5]. The clamor for locally manufactured antivenoms seems justified [21].

In the context of African spitting cobras, Indian manufactured antivenom is indicated for *Naja nigricollis* and *Naja haje* envenomation while the Mexican manufactured antivenom is indicated for *Naja nigricollis*, *Naja haje*, *Naja pallida*, *Naja nubiae* and *Naja katiensis* [22]. Therefore, it may be inferred that the cross-neutralization of toxic proteins in *N. ashei* venom by Mexican manufactured antivenom was because the toxicity profile of *N. ashei* venom may be similar to the profile in *Naja pallida, Naja nubiae,* and *Naja katiensis* venoms but dissimilar to *Naja nigricollis* and *Naja haje* venoms.

How can the pharmacological findings in this study be explained by what is known about the composition of *N. ashei* venom? Hus and colleagues indicated that the most abundant proteins in *N. ashei* venom were cytotoxins (3FTxs; three-finger toxins) and svPLA_2_’s [8]. Other venom proteins include 5′N-Snake venom 5′-nucleotidase; SVMPs—snake venom metalloproteinases; CRISPs—cysteine-rich venom proteins; CVF—cobra venom factor; and VNGF—venom nerve growth factor [8]. svPLA_2_’s may be acidic or basic and are divided into groups IA, IIA, and IIB [23]. The fact that this study used a pH of 8.1 to run the agarose-egg yolk assay strongly suggests that the observed svPLA_2_ activity was basic. This corroborates the findings of a previous study which reported that a majority of *N. ashei* venom proteins were of low molecular weight and basic [8]. Group IA svPLA_2_’s are primarily found in elapids, although some have been reported in colubrids [23]. Group IIA and IIB svPLA_2_’s are exclusively found in viperids [23]. Since *N. ashei* is an elapid, the svPLA_2_ activity observed was most likely of the Group IA variety.

*Naja ashei* venom exhibited strong cytotoxic action in the brine shrimp lethality assay relative to vincristine sulphate (a standard cytotoxic). It is important to note that the brine shrimp lethality assay is a good predictor of cytotoxicity and has been widely used to reliably detect this phenomenon in the venom of the sea snake; *Enhydrina schistosa* [24], and in several venomous fish [25–28], snails [29–31], toads [32] and bees [33]. The dose and time-dependent brine-shrimp lethality observed may be a direct consequence of the non-enzymatic effects of cytotoxins i.e. paralysis, Ca^2+^ toxicity, and cell death [34]. However, it is unlikely that this observation was not supported by the enzymatic action of the basic Group IA svPLA_2_’s which have been known to cause organelle toxicity, hydrolysis of the lipid environments of cell membranes, and mitochondrial membrane disruption of the respiratory muscle [34–36]. An important finding in this study was that the concentration of *N. ashei* venom was not the only predictor of svPLA_2_ activity. This raises a pertinent question: what other factors may be involved in predicting this activity? It was also observed that low and intermediate doses of *N. ashei* venom produced similar svPLA_2_ activities and there was no difference in the brine shrimp mortalities caused by intermediate and high doses of the venom. This may suggest that the activities of these toxins remain fairly constant within a narrow range of venom doses.

It was established that both antivenoms were safe in brine shrimp. The evaluation of the safety profile of the test antivenoms was important because (i) snake antivenoms may cause both acute (anaphylactic/pyrogenic) and delayed (serum sickness) toxic manifestations in human envenomation [37], and (ii) the safety profile was key in informing the selection of antivenom aliquots to be used in the neutralization assay.

Based on protein estimation by Lowry’s method, it was established that Indian manufactured antivenom had a higher protein content than Mexican manufactured antivenom but was ineffective in neutralizing the toxic effects of *N. ashei* venom. Because both antivenoms are made up of immunoglobulin-binding fragments; F(ab)’s [22] and given the fact that Lowry’s method largely reports the aromatic acid (tyrosine and tryptophan) composition of proteins [38], it may be argued that these amino acids may not be involved in the recognition and neutralization of toxic venom proteins in *N. ashei* venom.

### Conclusions

The svPLA_2_ activity and toxicity of *N. ashei* venom remain fairly constant within a narrow range of venom doses. Commercially available antivenoms are generally safe but have limited efficacy in neutralizing the svPLA_2_ activity of *N. ashei* venom. Moreover, only Mexican manufactured antivenom cross-neutralizes toxic venom proteins in *N. ashei* venom. We recommend studies on the activities of other toxins in this venom and their neutralization by antivenom.

## Limitations

Snake venom is a complex mixture of toxins. This study only evaluated the snake venom phospholipases A_2_ activity and brine shrimp lethality of *N. ashei* venom. To fully understand the capacity of antivenoms to neutralize *N. ashei* venom, it may be necessary to evaluate other toxins in this venom.

## Data Availability

All data generated or analyzed during this study are included in this published article. [And its additional information files below]. Information on the snakes whose venom was used in this study (Table S1); 10.6084/m9.figshare.12562055.v1. Details on the snake antivenom used in this study (Table S2); 10.6084/m9.figshare.12562055.v1. Raw data of the absorbance values (660 nm) of different concentrations of bovine serum albumin (protein standard), *Naja ashei* venom, and antivenom; 10.6084/m9.figshare.12562136.v2. The data output from the analysis of variance (+Tukey’s post hoc test) to determine dose-dependent differences in the svPLA_2_ activity of *Naja ashei* venom; 10.6084/m9.figshare.12562163.v2. The data output from the simple linear regression analysis to determine the %vPLA_2_ activity of different concentrations of Naja ashei venom (Figure S1); 10.6084/m9.figshare.12562175.v3. The data output from the simple linear regression to determine the capacity of antivenom I and II to neutralize the svPLA_2_ activity of *Naja ashei* venom (Figure S2), and (Figure S3);10.6084/m9.figshare.12570617.v1, 10.6084/m9.figshare.12571100.v1. The data output from probit regression analysis to determine the toxicity of *Naja ashei* venom, and vincristine and the safety of antivenom in brine shrimp;10.6084/m9.figshare.12571547.v1, 10.6084/m9.figshare.12571283.v1, and 10.6084/m9.figshare.12571283.v3. The data output from analysis of variance (+Tukey’s post hoc test) to determine dose-dependent differences in *Naja ashei* venom-induced brine shrimp lethality; 10.6084/m9.figshare.12562199.v2. The data output from simple linear regression to determine the capacity of antivenom to neutralize *Naja ashei* venom-induced brine shrimp lethality 10.6084/m9.figshare.12570620.v2. The data output from analysis of variance (+Tukey’s post hoc test) to determine the differences in the mean protein content of *Naja ashei* venom and antivenom I and II 10.6084/m9.figshare.12573425.v1.

## Abbreviations

svPLA_2_: Snake venom phospholipase A_2_
BSLA: Brine shrimp lethality assay
LC_50_: Lethal concentration responsible for 50% mortality
MPC_50_: The minimum phospholipase concentration responsible for 50% response
EC_50_: The least amount of antivenom (in µL) required to neutralize 1 mg of venom
DNA: Deoxyribonucleic acid
BSA: Bovine Serum Albumin
UV: Ultraviolet
mM: Millimole
v/v: Volume by volume
µL: Microliter
µg/mL: Microgram per milliliter
mm: Millimeter
ELISA: Enzyme-linked Immunosorbent assay
PBS: Phosphate-buffered saline
CaCl_2_: Calcium chloride
WHO: World Health Organization
mg/mL: Milligram per milliliter
r^2^: Coefficient of determination
LD_50_: Median lethal dose
